# Ocular Pharmacological Profile of Hydrocortisone in Dry Eye Disease

**DOI:** 10.3389/fphar.2019.01240

**Published:** 2019-10-18

**Authors:** Claudio Bucolo, Annamaria Fidilio, Claudia Giuseppina Fresta, Francesca Lazzara, Chiara Bianca Maria Platania, Giuseppina Cantarella, Giulia Di Benedetto, Chiara Burgaletto, Renato Bernardini, Cateno Piazza, Stefano Barabino, Filippo Drago

**Affiliations:** ^1^Department of Biomedical and Biotechnological Sciences, School of Medicine, University of Catania, Catania, Italy; ^2^Center for Research in Ocular Pharmacology–CERFO, University of Catania, Catania, Italy; ^3^Analytical Department, University of Catania Consortium Unifarm, Catania, Italy; ^4^Ocular Surface and Dry Eye Center, Ospedale L. Sacco, University of Milano, Milano, Italy

**Keywords:** dry eye disease, inflammation, cornea, hydrocortisone, Sjögren syndrome

## Abstract

To investigate the ocular pharmacological profile of hydrocortisone (HC) using *in vitro* and *in vivo* models of dry eye disease. Rabbit corneal epithelial cells (SIRCs) were used to assess the effect of HC in two paradigms of corneal damage: hyperosmotic stress and scratch-wound assay. Dry eye was induced in albino rabbits by topical administration of atropine sulfate or by injection of concanavalin A (ConA) into the lacrimal gland. TNFα, TNF-related apoptosis-inducing ligand (TRAIL), IL-1β, and IL-8 were determined by ELISA or western blot in a corneal damage hyperosmotic *in vitro* model, with or without HC treatment. Inflammatory biomarkers, such as TNFα, IL-8, and MMP-9, were evaluated in tears of rabbit eye injected with ConA and treated with HC. Tear volume and tear film integrity, in both *in vivo* models, were evaluated by the Schirmer test and tear break-up time (TBUT). Ocular distribution of four formulations containing HC (0.001%, 0.003%, 0.005%, and 0.33%) was performed in the rabbit eye. Aqueous humor samples were collected after 15, 30, 60, and 90 min from instillation and then detected by LC-MS/MS. Hyperosmotic insult significantly activated protein expression of inflammatory biomarkers, which were significantly modulated by HC treatment. HC significantly enhanced the re-epithelialization of scratched SIRCs. Treatment with HC eye drops significantly reduced the tear concentrations of TNF-α, IL-8, and MMP-9 vs. vehicle in the ConA dry eye model. Moreover, HC significantly restored the tear volume and tear film integrity to levels of the control eyes, both in ConA- and atropine-induced dry eye paradigms. Finally, we demonstrated that HC crossed, in a dose-dependent manner, the corneal barrier when the eyes were topically treated with HC formulations (dose range 0.003–0.33%). No trace of HC was detected in the aqueous humor after ocular administration of eye drops containing the lowest dose of the drug (0.001%), indicating that, at this very low concentration, the drug did not pass the corneal barrier avoiding potential side effects such as intraocular pressure rise. Altogether, these data suggest that HC, at very low concentrations, has an important anti-inflammatory effect both *in vitro* and *in vivo* dry eye paradigms and a good safety profile.

## Introduction

Corticosteroids are potent anti-inflammatory drugs that exert their pharmacological effects by binding to the glucocorticoid receptor (GR), leading to the regulation of gene expression by transrepression (i.e., regulating transcriptional regulators such as NF-kB) (Bekhbat et al., 2017). Ophthalmic eye drops of corticosteroids such as dexamethasone, triamcinolone, fluocinolone, loteprednol, and prednisolone are generally used in clinical practice to manage several ocular conditions, such as dry eye disease (DED) (Pinto-Fraga et al., 2016), conjunctivitis (Ridolo et al., 2014), uveitis, diabetic macular edema (Caceres-del-Carpio et al., 2016), and postsurgery inflammation (Aptel et al., 2017; Wielders et al., 2018). Dry eye has a disparate etiology that includes, among others, endocrine, immune, and iatrogenic factors, which can modify tear film homeostasis leading to inflammation (Musumeci et al., 2008; Bucolo et al., 2011; Lessard et al., 2013; Moutsopoulos, 2014; Bron et al., 2017). DED was originally considered a disease elicited by a deficient volume and quality of the tears. The Dry Eye Workshop (DEWS) of the Tear Film Ocular Surface Society (TFOS) described DED as “a multifactorial disease of the tears and ocular surface that results in symptoms of discomfort, visual disturbance, and tear film instability with potential damage to the ocular surface. It is accompanied by increased osmolarity of the tear film and inflammation of the ocular surface” (Craig et al., 2017). The canonical approach to treat DED focuses on tear replacement with artificial tears or, alternatively, on preserving the patient’s tears by means of occlusion of the tear drainage system (Ervin et al., 2017). These approaches have been demonstrated to decrease symptoms and signs of dry eye (Pucker et al., 2016), improving the resultant blurred vision and ocular discomfort (Liu and Pflugfelder, 1999). However, these treatments are considered palliative simply because they do not address the immuno-inflammatory process, that underlies the disease. Although the trigger of the pathogenic mechanism that leads to DED is not currently known, a growing body of evidence suggests that DED is characterized by an inflammatory process, which affects the lacrimal gland-ocular surface functional unit (Stern and Pflugfelder, 2004). The inflammation process feeds a vicious cycle of tear deficiency, contributing to ocular surface damage that in turn leads to symptoms and signs of chronic dry eye. Although artificial tears can have an indirect anti-inflammatory effect by lowering tear osmolarity and diluting pro-inflammatory factors on the ocular surface, they are not able to completely block this inflammatory vicious cycle. Ocular surface inflammation is crucial in the pathophysiology of dry eye, therefore anti-inflammatory therapy, including corticosteroids, may be of benefit to DED patients. Corticosteroids are prescription drugs for the treatment of DED along with antibiotics, integrin antagonists such as lifitegrast, and immunomodulatory drugs such as cyclosporine. In general, the use of prescription drugs for DED needs to be considered in the context of the single clinical history of the patient, along with the relative level of evidence supporting their use for that specific indication, as these drugs significantly differs in mechanism of action, and potential side effects. In spite of the remarkable anti-inflammatory properties of corticosteroids, chronic use often leads to undesired side effects, which are mainly related to the ability of the drug/receptor complex to bind specific response elements in the promoter regions of specific genes. In ophthalmic applications, chronic treatment of corticosteroids has been associated with side effects such as increased intraocular pressure (IOP) and increased risk of cataracts development (McGhee et al., 2002). Consequently, an unmet medical need exists for novel steroids formulations that promote remarkable anti-inflammatory activity, minimizing side effects. The use of topical “soft” steroids, such as loteprednol, has a lower likelihood of increasing IOP and inducing cataract formation (McGhee et al., 2002; Sheppard et al., 2016), even though these complications cannot be ruled out. Cortisol (called hydrocortisone, when used as a drug) is the main glucocorticoid with low anti-inflammatory action and short duration of action. Based on these premises, the purpose of the present study was to investigate the ocular pharmacokinetic/pharmacodynamics (PK/PD) profile of HC using *in vitro* and *in vivo* models of dry eye.

## Methods

### *In Vitro* Studies

#### Hyperosmotic Stress

Confluent rabbit corneal epithelial cells (SIRCs) at 16 days were exposed to an equal volume (0.5 ml/well) of serum-free medium (SHEM without FBS) for 24 h and then treated for 24 h with hyperosmolar media (450 mOsm/L) with or without 0.001% hydrocortisone treatment. The osmolarity value was chosen based on previous studies indicating a threshold of 450 mOsm/l for the induction of cell damage (Liu et al., 2009). The osmolarity of the culture media was assessed by an osmometer (Osmomat 30 Gonotech, Berlin, Germany). After 24 h, the levels of TNFα, TNF-related apoptosis-inducing ligand (TRAIL), IL-1β, and IL-8 were determined with ELISA kits (R&D System, Milan, Italy; RayBiotech, Milan, Italy) following the manufactures’ protocols. We also carried out a western blot assay to measure TRAIL expression in the cell lysates. Cells were harvested in RIPA lysis buffer supplemented with protease and phosphatase inhibitors cocktail (Sigma-Aldrich). After centrifugation at 14,000 rpm at 4°C for 10 min, the supernatants were collected. The total protein concentration in the supernatant was determined using the Bradford reagent (Bio-Rad Laboratories, Segrate, Italy) and measuring absorbance with a VarioskanTM Flash Multimode Reader. Equal amounts of protein (30 μg) were resolved by 8–12% (10%) SDS-PAGE and then transferred to Hybond ECL nitrocellulose membranes (GE Healthcare, Little Chalfont, UK). Membranes were blocked with 5% nonfat dry milk in phosphate-buffered saline plus 0.1% Tween 20 (PBS-T) (Bio-Rad Laboratories, Segrate, Italy) and then incubated overnight at 4°C with rabbit anti-TRAIL/TNFSF10 polyclonal antibody (Abcam, cat. No. ab2435; 1:200). The membranes were then washed with PBS-T and finally probed with horseradish peroxidase-conjugated antirabbit IgG secondary antibody (GE Healthcare, cat. No. GENA934; 1:5000) for 1 h at RT in 5% nonfat dry milk. Detection of specific bands was carried out using the iBright Imaging Systems (Thermo Fisher Scientific, Inc.) after enhanced chemiluminescence (ECL) (GE Healthcare). β-actin (Santa Cruz Biotechnology, sc-47778; 1:1000) was used as the housekeeping protein. Densitometric analysis of band intensity was carried out by the ImageJ software (https://imagej.nih.gov/ij/). All experiments were repeated at least four times, each run in triplicate.

#### Scratch-Wound Assay

A scratch-wound assay on SIRCs was used to assess the effects of HC on wound areas. SIRCs were purchased from ATCC^®^ and cultured in Eagle’s minimum essential medium (EMEM, Sigma-Aldrich) with 10% of FBS (fetal bovine serum), 1X MEM NEAA (minimum essential medium nonessential amino acids) and 1X P/S (penicillin/streptomycin). Cellular adhesion was promoted applying 5–10 µl gelatin solution/cm^2^ (i.e., 0.1–0.2 mg/cm^2^ gelatin) as plate coating agent. Cells were incubated at 37°C in 5% CO2 in humidified air. Cells were seeded (1.7 × 105) on 24-well culture plates (Corning^®^) and cultured until confluence, as previously described. After confluence, cells were washed twice with warm phosphate saline buffer (PBS, 1X) and then incubated with serum-free medium for 5 h (starvation). Then, the SIRC monolayer was scratched with a sterile 200 µl pipette tip, and the wells were washed with fresh medium to remove detached cells before incubation in serum-free medium containing sodium hyaluronate 0.2% and HC (0.001%). Pictures of the wound areas were taken and the coordinates noted at starting time of experiment (T0) and forty-eight hours (T48) after scratch. T0 and T48 wound images were obtained with an inverted optical microscope (IM3 Optika), 20X magnification. Four images per condition were analyzed and mean wound area was measured with the image analysis system Image J Software (https://imagej.nih.gov/ij/). We reported the percentage of wound closure, as normalized to the corresponding wound area at T0.

### *In Vivo* Studies

#### Animals

Male New Zealand albino rabbits (1.8–2.0 kg) were purchased from Envigo (Udine, Italy). Animals were housed under standard conditions with food and water provided *ad libitum* in a light-controlled room and set temperature and humidity. Animal care and experimental procedures were carried out according to the ARVO Statement for the Use of Animals in Ophthalmic and Vision Research. Protocols were approved by the Institutional Animal Care and Use Committee of the University of Catania (project #303).

#### Ocular Distribution

Four eye drops containing hydrocortisone (FA = 0.001%, FB = 0.003%, FC = 0.005%, and FD = 0.33%) and sodium hyaluronate (0.2%) were administered in the conjunctival sac four times every 2 h into the rabbit eye. Aqueous humor samples (n = 4 for each time point) were collected after 15, 30, 60, and 90 min from the last instillation and hydrocortisone detected by liquid chromatography tandem-mass spectrometry (LC-MS/MS). The internal standard prednisolone (5 μl of 2 μg/ml solution) was added to 100 μl of aqueous humor samples. Ethyl acetate was then added, and the samples were vortexed, centrifuged (12,000 rpm 25 min 4°C), and dried. After drying, 100-μl HPLC mobile phase was added to samples, subjected to vortexing and spinning. Samples were then injected into LC-MS/MS (Agilent 6410A Triple Quadrupole). We determined the limit of detection method, which was 2.5 ng/ml for HC. The data generated by the present study were used to choose the right HC concentration to be studied for the evaluations of pharmacodynamics.

#### Dry Eye Models

Two different paradigms were used to investigate the ocular pharmacodynamics profile of hydrocortisone: aqueous tear deficiency model and lacrimal gland inflammation-induced dry eye model elicited by atropine and concanavalin A (ConA).

Atropine sulfate (1.0%) eye drops (Atropina Farmigea, Farmigea, Pisa, Italy) was instilled into the lower conjunctival sac of the eye (four times within 12 h). Atropine is a muscarinic (M_3_) receptor antagonist, which is able to decrease tear volume and alter tear film stability. Fifteen minutes after atropine administration, we treated the eyes with 0.001% HC eye drops or vehicle (four times within 12 h). To assess the degree of dry eye after 24 h from the atropine instillation, tear volume and tear film breakup time (TBUT) were used as endpoints. Tear volume was evaluated by Schirmer strips (Eagle Vision, Memphis, TN, USA). The strips were carefully placed in the posterior (i.e., temporal) lower fornix for 60 s, and the wetted area was evaluated. TBUT was assessed after instillation (5 µl) of sodium fluorescein (2%) in the rabbit eye. A slit lamp (Sbisà, Firenze, Italy) with cobalt blue filter was used to enhance fluorescein patterns; TBUT was determined by measuring the time from the opening of the eyes until the appearance of the first black spot or streak on the cornea. The procedure was sequentially performed three times and the average of three readings per eye was calculated. Data were analyzed by two investigators who were unaware of the experimental design. Two groups of animals (n = 6/group) were used and treated as follows: (1) control group (atropine treatment + eye drops vehicle) and treated group (atropine treatment + 0.001% HC).

A separate set of rabbits was treated with the T-cell mitogen ConA (300 μg/50 μl of BSS) in the lacrimal glands using a 28-gauge needle. Before ConA injection, the animals were anesthetized by dexmedetomidine (20 µg/kg intramuscularly; Dexdomitor™,Vetoquinol, Bertinoro, Italy) and tiletamine + zolazepam (10 mg/kg intramuscularly; Zoletin™, Virbec, Milan Italy). Control rabbits were injected with 50 μl of BSS in the lacrimal gland. Rabbits were randomized into two treatment groups (n = 5 per group) and instilled with ophthalmic solution four times a day for 3 days with either drug (0.001% HC) or vehicle. After 3 days from ConA injection, tears were collected and TNF-α, IL-8, and MMP-9 were measured following the manufactures’ protocols (R&D System, Milan, Italy; RayBiotech, Milan, Italy). A separate set of animals was used to assess the safety profile, in terms of intraocular pressure, of the ophthalmic formulation with the lowest concentration of the drug (0.001% HC). One drop was instilled into the conjunctival sac (four times within 12 h for 4 weeks), and IOP measured every week by TonoPen (Reichert, Buffalo, NY, USA).

### Statistical Analysis

The data were expressed as mean ± SD. Statistical analysis was conducted using ANOVA followed by Dunnett’s test. Student’s t-test was performed where appropriate. A *p*-value *<* 0.05 was predetermined as the criterion of statistical significance.

## Results

### *In Vitro* Studies

**Figure 1:**
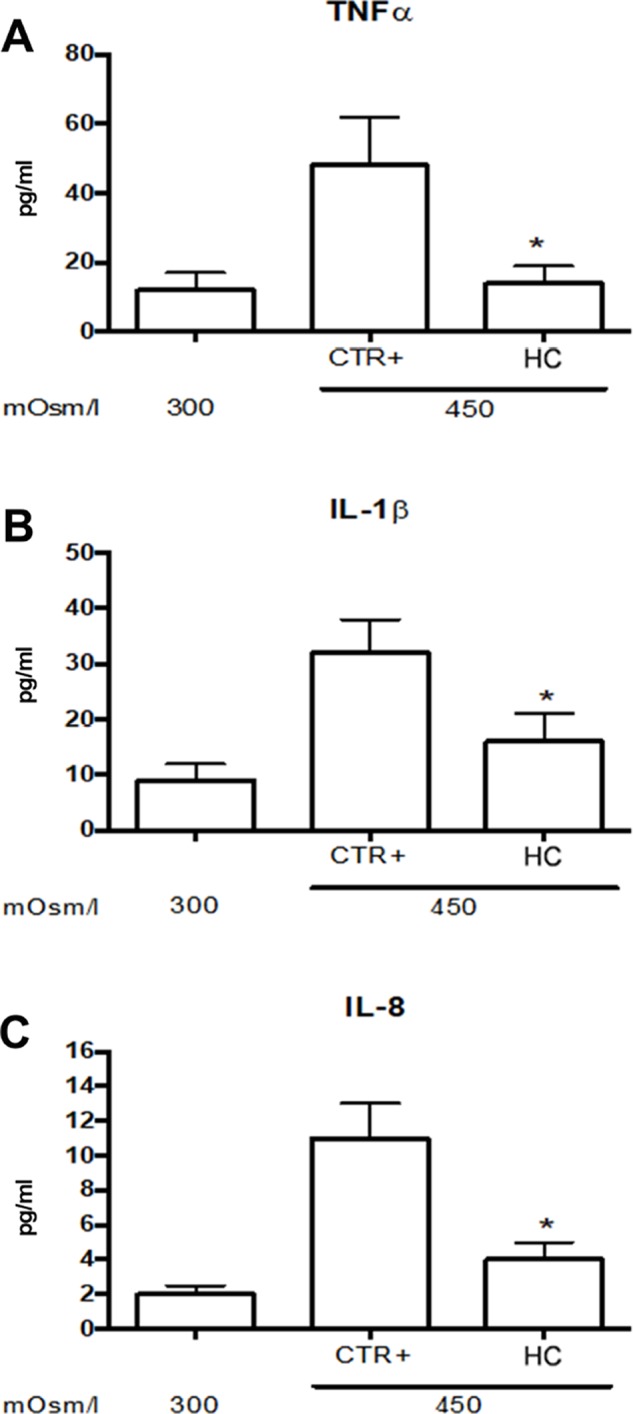
Inflammatory cytokine levels in SIRCs exposed to hyperosmotic stress. **(A)** TNFα **(B)** IL-1β and **(C)** IL-8 levels.*p < 0.05 HC treatment vs. Ctrl+ (SIRCs with hyperosmotic insult); SIRCs, rabbit corneal epithelial cells; HC, hydrocortisone; n = 6.

Tear film osmolarity is an important causative factor in the pathogenesis of DED. Tear hyperosmolority resulting from decreased lacrimal flow contributes to ocular surface damage through a cascade of inflammatory events. For this reason, we used an *in vitro* paradigm that mimics this clinical condition. We induced corneal cell damage by a hyperosmotic environment and assessed the effects of a low concentration (0.001%) of HC on cytokine expression. The corneal cells exposed to hyperosmotic insult for 24 h showed a significant (p < 0.05) increase of TNFα, IL-1β, and IL-8 levels that were significantly (p < 0.05) counteracted by HC treatment (**Figure 1**).

**Figure 2:**
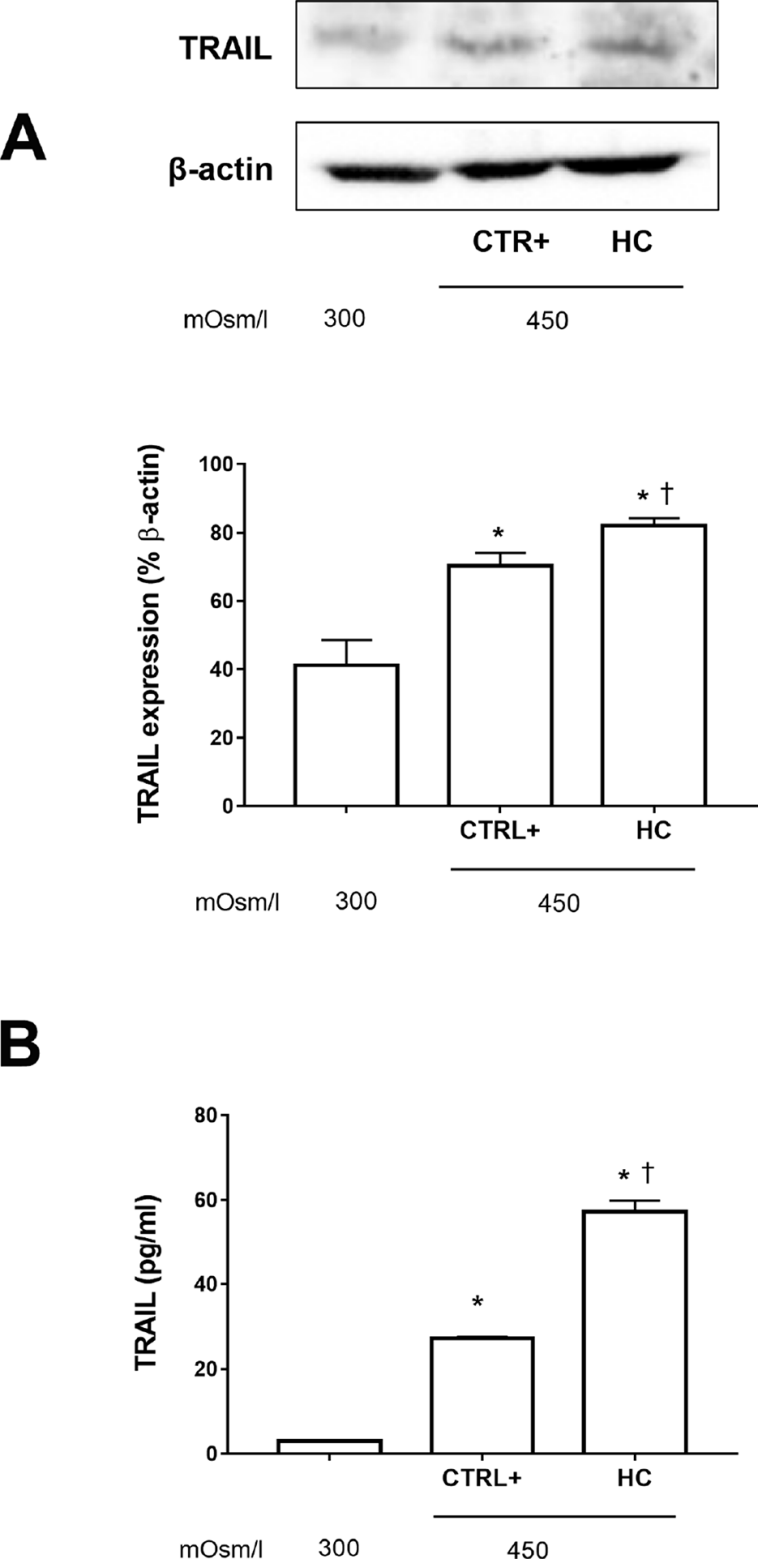
TRAIL levels in SIRCs exposed to hyperosmotic stress. **(A)** Western-blot analysis of SIRCs lysates for TRAIL expression. **(B)** ELISA quantification of TRAIL in SIRCs medium. *p < 0.05 vs. SIRCs growth in medium 300 mOsm, ^†^p < 0.05 vs. CTRL+ (SIRCs with hyperosmotic insult); SIRCs, rabbit corneal epithelial cells; HC, hydrocortisone; n = 6.

**Figure 3:**
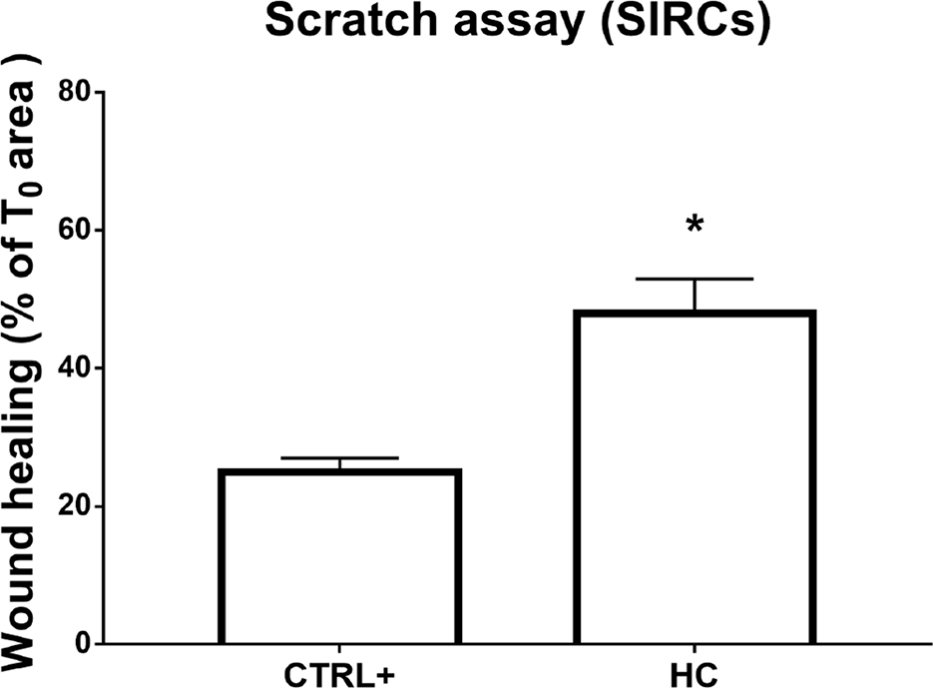
Wound healing in SIRCs monolayer. *p < 0.05 HC treatment vs. CTRL+ (SIRCs monolayer scratch); SIRCs, rabbit corneal epithelial cells; HC, hydrocortisone; n = 6.

We assessed TRAIL expression in SIRCs exposed to a hyperosmolarity condition w/o 0.001% HC (**Figure 2**). Hyperosmotic insult increased significantly (p < 0.05) the expression of TRAIL in SIRCs, compared to control cells (300 mOsm). Furthermore, hydrocortisone treatment significantly (p < 0.05) increased TRAIL expression compared to SIRCs exposed to hyperosmotic stress (**Figure 3A**). In order to assess if TRAIL levels in the SIRC medium were in the protective range (≤10 ng/ml), we measured soluble TRAIL by ELISA. Medium TRAIL content in SIRCs (control, 300 mOsm) was very low and increased with either a hyperosmotic insult (27 pg/ml) or HC treatment (57 pg/ml) that means five logs below the toxic cutoff (100 ng/ml; Lee et al., 2002) (**Figure 2B**).

Furthermore, the wound healing assay was performed on the corneal cell monolayer. Migration ability was assessed in a scratch assay on the SIRC monolayer. Microscopic bright field pictures were taken, and wound areas were evaluated at 48 h and normalized to areas at T0. HC at 0.001% displayed a statistically significant (p < 0.05) effect on wound healing compared to controls (**Figure 3**). Indeed, HC at the tested concentration did not influence the wound healing of damaged corneal epithelial cells.

### In Vivo Studies

#### Ocular Distribution

**Figure 4:**
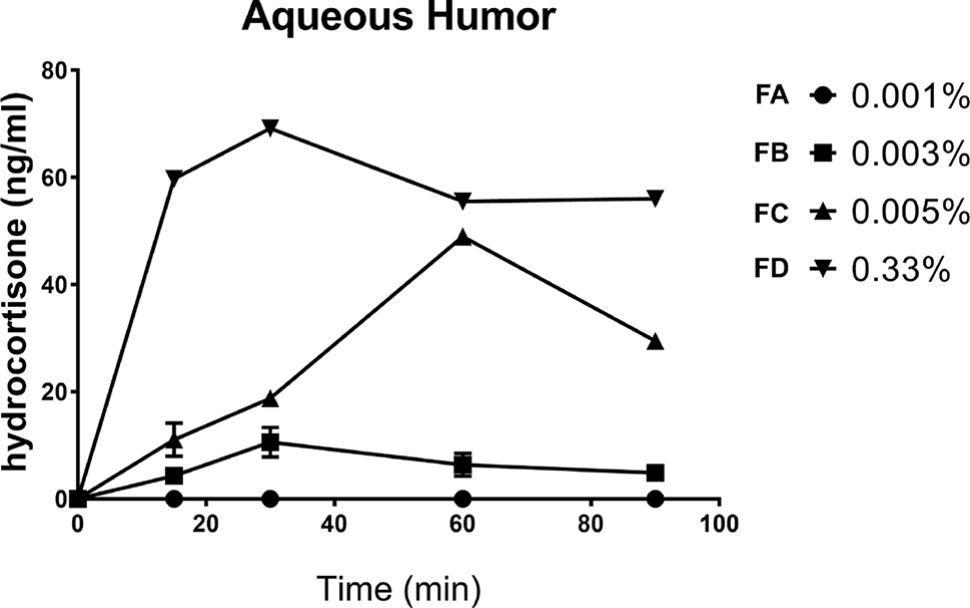
Aqueous distribution of hydrocortisone after instillation of four formulations in the rabbit eye.

This study was important in order to choose the right dose to carry out the pharmacodynamics study. We determined the PK profile of four HC eye drops, tested at four different concentrations (FA = 0.001%, FB = 0.003%, FD = 0.005%, and FC = 0.33%). No levels, at any time, of hydrocortisone were detected in the aqueous humor in the group treated with the formulation (FA) containing the lowest drug concentration (0.001%). On the contrary, the aqueous humor samples obtained from the other three groups (0.003%, 0.005%, and 0.33%) showed remarkable levels of hydrocortisone (**Table 1**, **Figure 4**). We demonstrated that HC crosses the corneal barrier, in a dose-dependent manner (see the C_max_ and AUC_0-90_ values, **Table 1**) Our data confirmed that HC at the lowest dose (0.001%) does not cross the corneal barrier; therefore, the risk of side effects, such as intraocular pressure rise, is negligible. This allowed us to choose the right HC concentration to be studied for the evaluations of efficacy.

**Table 1:** Aqueous distribution of hydrocortisone after instillation of four formulations in the rabbit eye: PK parameters.

% w/v hydrocortisone	C_max_ (ng/ml)	T_max_ (min)	AUC_0–90’_ (ng·min/ml)
0.001%	–	–	–
0.003%	10.6 ± 2.8	30	535.1 ± 65.9*
0.005%	48.94 ± 1.14	60	2417 ± 50*
0.33%	69.15 ± 1.00	30	4510 ± 30*

*p < 0.05 for TWO-WAY ANOVA comparison between treatment groups.

#### *In Vivo* Efficacy Study

In the atropine DED animal model, we assessed tear volume and tear film quality after 0.001% HC treatment, with Schirmer’s test and TBUT, which are currently used in clinical practice to diagnose dry eye and assess severity grade (Barabino et al., 2004).

**Figure 5:**
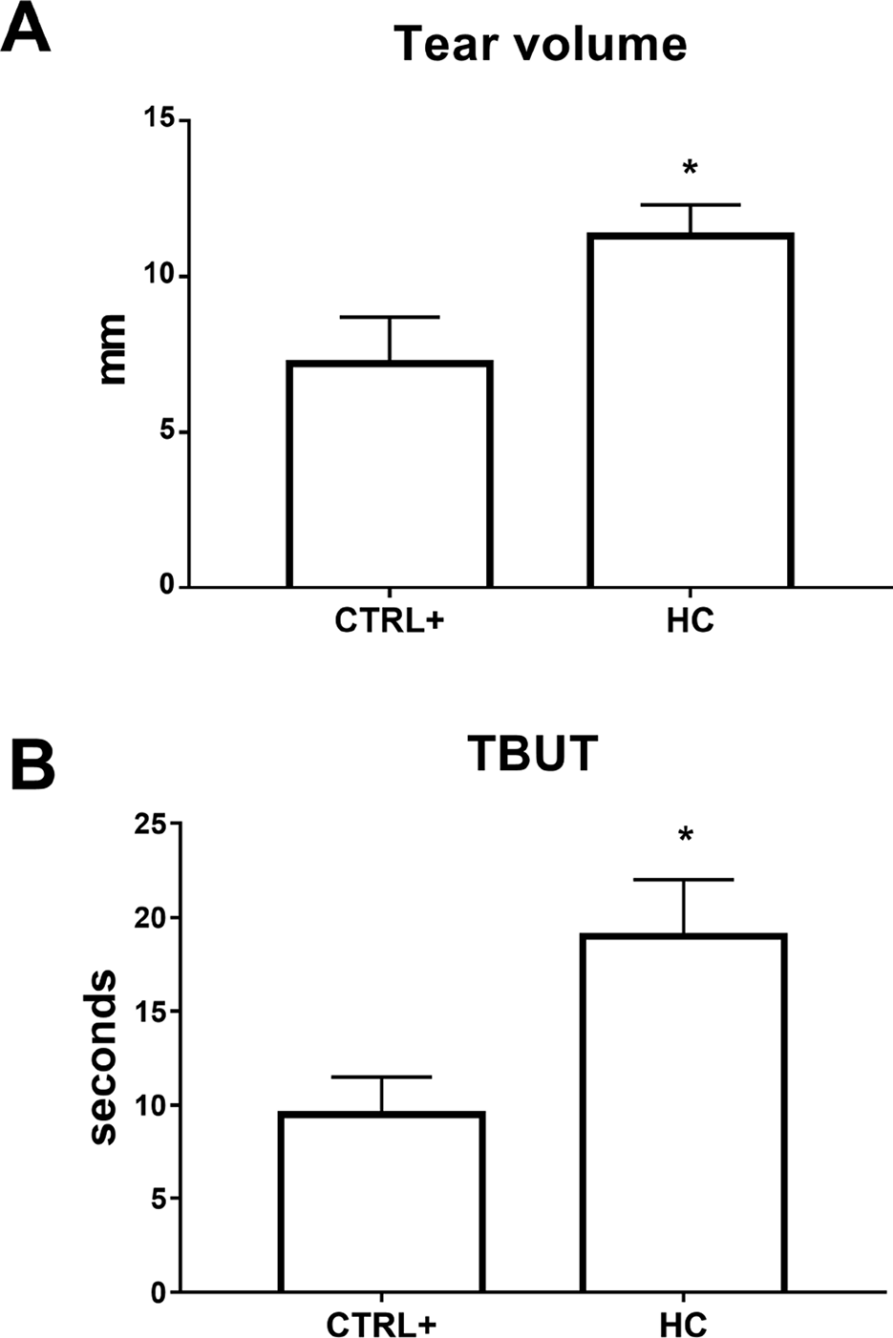
Tear volume **(A)** and tear film stability **(B)** in atropine induced dry eye model. TBUT, tear breakup time. *p < 0.05 HC vs. CTRL+ (eye treated with atropine; no treatment with HC); HC, hydrocortisone; n = 6.

Tear volume and tear film integrity were measured, before (13.5 ± 0.5 mm and >60 s, respectively) and after 24 h from the first atropine instillation, by Schirmer’s test and TBUT, respectively. The baseline values of tear volume and TBUT were in accordance with previous study (Shafiee et al., 2011). Multiple topical administrations of atropine reduced tear volume (around 5 mm) in the vehicle-treated group; this effect was significantly (p < 0.01) counteracted by topical treatment with hydrocortisone (0.001%) eye drops (**Figure 5A**). A similar trend was also observed in terms of tear film integrity as measured by TBUT. Vehicle-treated eyes showed a marked and immediate reduction in TBUT that started at day 1 (reduction of TBUT to around 10 s) and continued at day 3 (TBUT around 8 s) (**Figure 5B**). In contrast, HC eye drops significantly (p < 0.01) preserved the tear film integrity at 24 h from atropine instillation (**Figure 5B**). Finally, repeated topical administration of the ophthalmic formulation at the lowest dose of HC showed no change in terms of IOP values throughout the 4 weeks (data not shown).

**Figure 6:**
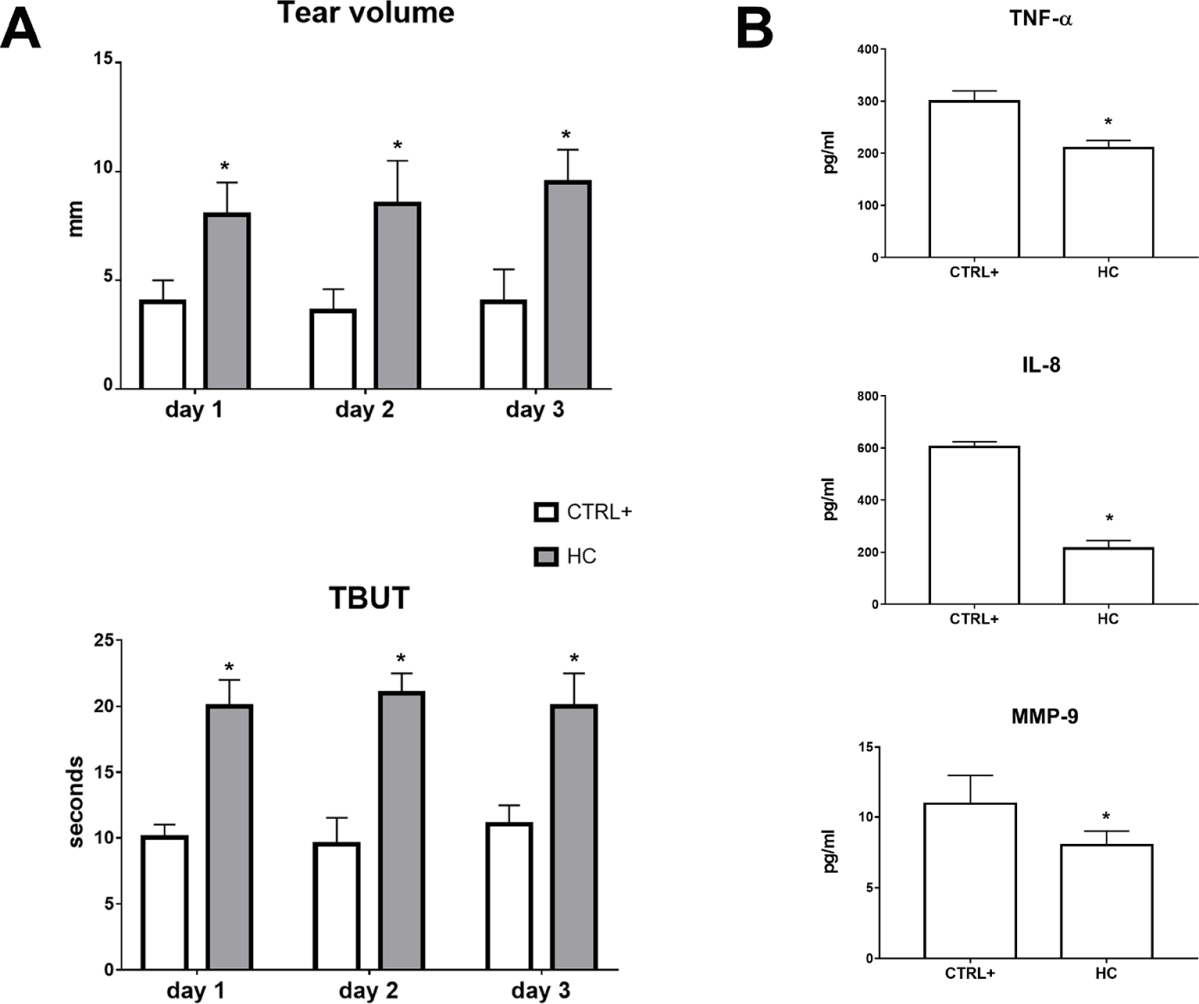
Tear volume, tear film stability **(A)**, and inflammatory biomarkers **(B)** in ConA induced dry eye model. TBUT, tear breakup time. *p < 0.05 HC vs. CTRL+ (eye treated with atropine; no treatment with HC); HC, hydrocortisone; ConA, concanavalin A; n = 6.

A lacrimal gland inflammation-induced dry eye model was used in a separate set of animals. Rabbits received a ConA (T-cell mitogen) injection into the lacrimal gland. Tear volume and tear film quality were affected in the ConA animal model of DED and 0.001% HC treatment reverted tear volume and TBUT to control levels (CTRL+ = injected with ConA; no treatment with HC) (**Figure 6A**). After 3 days, inflammatory biomarkers were measured in the tear samples. Topical treatment with hydrocortisone (0.001%) significantly (p < 0.05) reduced the tear levels of TNF-α, IL-8, and MMP-9 compared with the vehicle group. Topical treatment with hydrocortisone (0.001%) significantly (p < 0.05) counteracted the tear volume reduction and tear integrity at all the time points (**Figure 6B**).

## Discussion

The production of tears is regulated to maintain tear osmolarity within narrow limits at all times (Sullivan et al., 2012). Tear film osmolarity dysregulation is one of the key factors in the pathogenesis of DED, including Sjögren syndrome, a chronic autoimmune disorder. Tear hyperosmolarity, resulting from decreased lacrimal flow or tear film instability, contributes to ocular surface damage both directly and, indirectly, through a cascade of inflammatory events. Physiological levels of tear film osmolarity are in the range of 302 ± 9.7 mOsm/l (Tomlinson et al., 2006). Meanwhile, the highest values for tear osmolarity measured in DED patients are below 500 mOsm/l. However, it is likely that the levels achieved at the ocular surface are much higher than 500 mOsm/l, particularly at the cornea sites where tear film starts to breakup.

Low aqueous tear flow and/or excessive evaporation induce hyperosmolarity, eliciting the release of inflammatory mediators into the tear fluid. Clinical studies consistently report upregulation of several pro-inflammatory mediators in the tears of DED patients such as interleukins, cytokines, and metalloproteinases (Lin et al., 2010; Zhang et al., 2011). The last group is composed by endopeptidases involved in tissue remodeling, and elevated levels of MMP-9 have been shown to be diagnostic in clinical evaluations of DED. It is well known that desiccating and hyperosmolarity stresses induce expression of NFκB, which is involved in signaling and subsequent inflammatory alterations related to DED. The NFκB signaling pathway regulates the expression of cytokines and MMPs, such as MMP-9, which is involved in the pathogenesis of DED.

In general, inflammation is an adaptive response of the immune system to noxious insults to maintain homeostasis and restore functionality. Recently, a new concept of the origin of inflammation suggested that, between basal homeostatic conditions and canonical inflammation, there is a condition called “para-inflammation”(Medzhitov, 2008). Para-inflammation is an adaptive response of the immune system to low levels of tissue damage (i.e., a low degree of “danger” stimuli), such as in DED where hyperosmotic stress accumulates bit by bit for years. The physiological role of para-inflammation is to maintain homeostasis (or reset the homeostatic threshold of the tissue) and restore tissue functionality, even though para-inflammatory processes could be dysregulated, contributing to corneal damage. Therefore, the new concept is that a well-controlled para-inflammation is beneficial, while a dysregulated para-inflammation is detrimental. In this context, the protein TRAIL plays an important role in the modulation of autoimmune inflammation. TRAIL is a protein known for its ability to promote cell death and is constitutively expressed in ocular tissues including the cornea (Lee et al., 2002; Narayanan et al., 2006). However, several groups have recently shown that TRAIL can also stimulate cell proliferation (Kavurma et al., 2008; Chan et al., 2010), with anti-inflammatory effects (Manuneedhi Cholan et al., 2018). Indeed, these reports suggest a protective role of TRAIL, even though the precise mechanism/s are not clear yet. This dual effect could be related to several factors such as, among others, different *milieu*, diverse insults, and various grades of damage.

In the present study, we observed an increase of inflammatory biomarkers such as TNF-α, IL-1β and IL-8 in corneal cells after hyperosmotic stress that were attenuated by HC treatment at a very low concentration. Furthermore, we found that TRAIL expression was increased in corneal cells exposed to hyperosmotic stress also in the presence of very low concentrations of HC; interestingly, the TRAIL levels detected were in a concentration range known to be protective (≤10 ng/ml) (Hilliard et al., 2001; Cantarella et al., 2014; Di Bartolo et al., 2015; Cartland et al., 2016). These findings support the hypothesis that a corneal physiological mechanism of defense is triggered when the cells are undergoing osmotic insult and that this protective pathway is facilitated by HC. It is noteworthy that TRAIL controls autoimmune inflammation, and Hilliard et al. (2001) demonstrated that TRAIL inhibits experimental autoimmune encephalomyelitis by preventing activation of autoimmune T cells (Hilliard et al., 2001). This point is important considering that T cells play a major role in the detrimental inflammatory process that occurs in the lacrimal glands of Sjögren syndrome patients (Moutsopoulos, 2014).

In summary, our *in vivo* data demonstrate that 0.001% HC eye drops are an effective anti-inflammatory therapy when tested in an aqueous tear deficiency model (atropine-induced dry eye) and lacrimal gland inflammation-induced dry eye model (ConA-induced dry eye) in rabbits. The present study demonstrated, both *in vitro* and *in vivo*, that the anti-inflammatory action of HC on the ocular surface is related to the modulation of TNFα, TRAIL, IL-1β, IL-8, and MMP-9. The quenching action of HC on the inflammatory process brought about a restoration of ocular tissues, tear film as demonstrated, among others, by Schirmer’s test and TBUT. Finally, the pharmacokinetics study demonstrated that after topical administration of the ophthalmic formulation, containing the lowest dose of HC (0.001%), in the rabbit eye, no trace of the drug was detected in the aqueous humor. Altogether, these data suggest that hydrocortisone, at a very low concentration, has a relevant anti-inflammatory effect on both *in vitro* and *in vivo* dry eye models, with a good impact in terms of safety, warranting further clinical evaluation.

## Data Availability Statement

All datasets generated for this study are included in the manuscript/supplementary files.

## Ethics Statement

The animal study was reviewed and approved by University of Catania.

## Author Contributions

Authors that made substantial contributions to conception and design: CB, SB, and FD. Authors that made contribution to acquisition of data: CB, AF, CGF, FL, CBMP, GC, GB, CBurg, and CP. Authors that made contribution to statistical analysis and interpretation of data: CB, CBMP, GC, and RB. Authors that participated in drafting the article and revising it critically: CB, GC, RB, SB, and FD.

## Conflict of Interest

The authors declare that the research was conducted in the absence of any commercial or financial relationships that could be construed as a potential conflict of interest.
